# Gastrointestinal parasites in West African chimpanzees (*Pan troglodytes verus*) in Fongoli (Kedougou, Senegal)

**DOI:** 10.5194/pb-12-15-2025

**Published:** 2025-12-19

**Authors:** Papa Mamadou Sy, Kacou Martial N'da, Papa Ibnou Ndiaye, Oubri Bassa Gbati, Jill Daphne Pruetz

**Affiliations:** 1 Département de Biologie Animale, Faculté des Sciences et Techniques de l'Université Cheikh Anta Diop, Dakar, BP 5005, Senegal; 2 Département Santé Publique-Environnement, Ecole Inter-Etats des Sciences et Médecine Vétérinaires de Dakar, BP 5077, Senegal; 3 Observatoire Hommes-Milieux International Téssékéré; Laboratoire de Recherche International (IRL3189 ESS) Environnement, Santé et Sociétés, Université Cheikh Anta Diop, Dakar, Senegal; 4 Department of Anthropology, Texas State University, San Marcos, TX, USA

## Abstract

Natural ecosystems are severely disrupted by human activities. Our interactions with wildlife are intensifying and promoting zoonosis. Humans and chimpanzees can harbour and transmit pathogens to each other. The aim of this study is to improve our knowledge of the diversity of gastrointestinal parasites in Fongoli chimpanzees. This is a habituated group that has been monitored over the long term and whose members have all been identified. During the period from 22 February to 11 March 2022, we monitored them daily to collect fresh stool samples in a non-invasive manner. A total of 17 individuals were sampled for 39 faeces samples collected and fixed in 10 % formalin. In the laboratory, we performed a coproscopical analysis of the fixed faeces using flotation and sedimentation methods. The parasite diversity included six protozoa (*Troglodytella* spp., *Troglocorys* spp., *Entamoeba coli*, *Entamoeba* spp., an unidentified ciliate, and Coccidia) and six helminths (*Enterobius* spp., *Strongyloides* spp., *Dicrocoelium* spp., *Ascaris* spp., Spirurids, and Strongylids). We found protozoa in all individuals and helminths in 70 % of individuals. We found an average of 6 ± 1.41 types of gastrointestinal parasites, including 1.47 ± 1.07 helminths per individual. Chimpanzees in Fongoli harbour a significant diversity of intestinal parasites, some of which are common to humans and have zoonotic potential.

## 1 Introduction

Advances in technology and global population growth have increased pressure on natural resources. Deforestation and habitat fragmentation are among the major causes of biodiversity loss (Morand and Lajaunie, 2021). Interactions between humans and wildlife are becoming more frequent as we transform natural habitats for urbanisation and subsistence needs (Devaux et al., 2019). This proximity promotes the mutual transmission of zoonotic diseases (Patz et al., 2000; Faraldo and Rebaudet, 2020). Approximately 75 % of emerging infectious diseases affecting humans originate in wildlife (Faraldo and Rebaudet, 2020). Anthropogenic disturbances can alter the behaviour of wildlife, which then seeks to adapt to new environmental conditions. Non-human primates are a good example of this, as they raid agricultural areas or visit human waste dumps in forests (Gilardi et al., 2016; McLennan et al., 2017). Some of them, such as great apes, remain vulnerable to these disturbances because they depend on wooded habitats for shelter and food. The chimpanzee (*Pan troglodytes*), considered the most flexible of the great apes, has nevertheless experienced a significant decline in population size due to anthropogenic pressures and habitat loss (Humle et al., 2016; Kühl et al., 2017).

In southeastern Senegal, the majority of the western chimpanzee (*Pan troglodytes verus*) population lives outside protected areas (Ndiaye et al., 2018; Heinicke et al., 2019), with their habitats often overlapping with areas of human occupation. Human and livestock encroachment on their habitats occurs through industrial and artisanal gold mining, timber trafficking, and grazing (Badji, 2019; Diallo et al., 2022). Chimpanzees can be affected by viral, bacterial, and parasitic infections (Gilardi et al., 2016, 2025), which they can transmit to humans due to the phylogenetic proximity between our two species (Barriel, 2004; Calvignac-Spencer et al., 2012). Chimpanzees are also vulnerable to diseases originating in humans, particularly respiratory infections, which are often associated with high mortality rates (Carter et al., 2003; Chapman et al., 2005; Gilardi et al., 2016, 2025). Disorders caused by parasitic diseases, in this case helminthiasis and protozoosis, may be perceived as less of a concern than serious viral and bacterial infections (Gilardi et al., 2016). However, they can reduce the host's condition and alter its nutritional status. Chimpanzees can tolerate certain macroparasites when they are in good condition and are able to develop self-medication behaviours to regulate the populations of these parasites (Krief et al., 2003; Pruetz and Johnson-Fulton, 2003). This ability would depend on the quality of the resources available in their habitats. Thus, the reduction in habitats and resources following anthropogenic disturbances can have stressful and harmful effects on the health and resistance of individuals (Coop and Holmes, 1996; Chapman et al., 2006). Consequently, infestations that were previously well tolerated may result in higher morbidity and mortality (Gillespie and Chapman, 2008).

The study of gastrointestinal parasites allows non-invasive health monitoring of wild primates (Gillespie, 2006; Howells et al., 2011), especially in areas such as Fongoli where chimpanzees coexist with humans and domestic animals. The Fongoli chimpanzee group is currently facing increasing habitat degradation, compromising its long-term survival (Jill D. Pruetz, personal communication, 2024). Several parasite surveys have been conducted on this group, the first of which took place before the intensification of human activities, particularly artisanal gold mining (Howells et al., 2011). Thus, an increase in potentially zoonotic parasitic infestations is reasonably expected given the current context of anthropisation. This study therefore aims to determine the diversity and prevalence of gastrointestinal parasites in Fongoli chimpanzees for comparison with previous assessments.

## 2 Materials and methods

### 2.1 Study area

**Figure 1:**
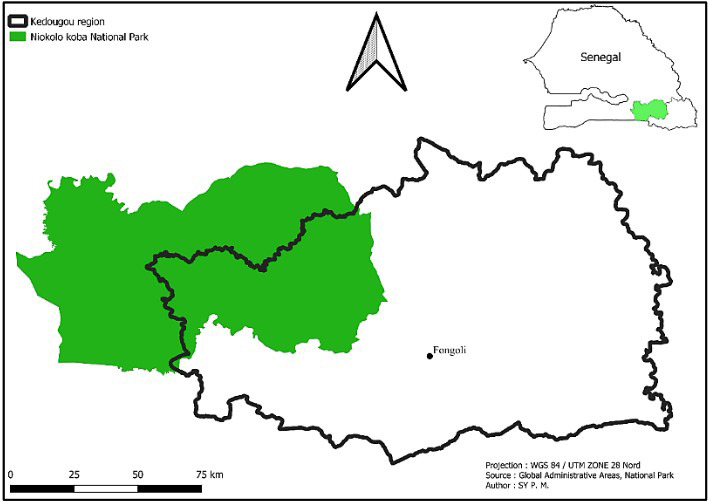
Location of the study site.

Our study area is located near the hamlet of Fongoli, in the Bandafassi district of the Kedougou region (Fig. 1). It has a Sudano-Guinean climate, with a rainy season from May to October and annual rainfall of around 1000 mm yr^−1^ (ANACIM, 2022). During the dry season (November–April), temperatures vary considerably depending on the month, with average maximum temperatures between 31–42° and average minimum temperatures between 19–28° (ANACIM, 2022). The region is characterised by various types of habitats, mainly consisting of grasslands, shrub savannahs, wooded savannahs, and a few pockets of gallery forests (McGrew et al., 1981; Pruetz and Johnson-Fulton, 2003). This mosaic savannah is also very rich in flora including *Pterocarpus erinaceus*, *Khaya senegalensis*, *Diospyros mespiliformis*, *Bambusa vulgaris*, and *Combretum* sp. This resource is exploited by other sympatric animal species, including non-human primates such as baboons, green monkeys, and galagos and other mammals such as warthogs and bushbuck. It is also exploited by humans through grazing, gathering, agriculture, and gold panning (Pruetz, 2006; Lindshield et al., 2019). It is therefore an area where frequent interactions between humans, domestic animals, and wildlife can contribute to the circulation of zoonotic pathogens.

**Figure 2:**
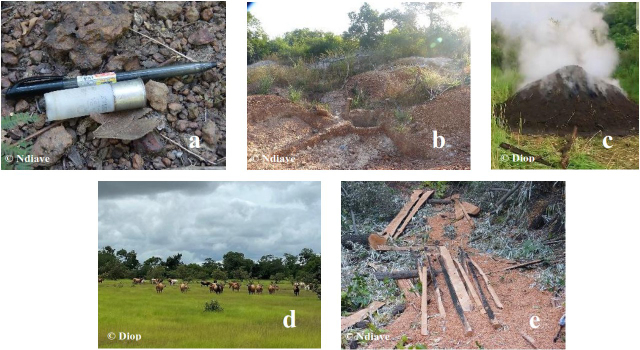
Human activities present in the habitat of chimpanzees at Fongoli: **(a)** hunting, **(b)** gold panning, **(c)** charcoal, **(d)** pastoralism, **(e)** deforestation.

The human population of the Fongoli hamlet is reduced to one resident family, artisanal gold mining workers, and researchers. Gold mining and its connected human activities are growing progressively in the Fongoli area. Many disturbances of the chimpanzee's habitat in relation to anthropogenic activities and development infrastructures are described (Fig. 2), namely gold mining, poachers, agriculture, line power, and road construction near to the chimpanzee's habitat in Kedougou (Pruetz and Herzog, 2017; Badji, 2019; Diallo et al., 2022).

### 2.2 The chimpanzee study group

The Fongoli chimpanzees constitute a habituated group whose members are individually identified. The group consists of 31 individuals, including 10 adult males, 8 young males, 9 adult females, and 4 young females during the study period. The size of their home range is estimated to be at least 63 km^2^ (Pruetz, 2006) but is expanding as research into their area of activity becomes more refined. Chimpanzees in Fongoli now have to adapt to numerous challenges, ranging from the aridity of the environment, high daytime temperatures, and scarce water sources during the dry season and recurrent bush fires (Pruetz, 2001; Pruetz and Herzog, 2017). Added to this is the degradation of their habitats due to human activities such as gold mining, timber trafficking, and agricultural activities (Badji, 2019; Diallo et al., 2022). Chimpanzees also compete with humans for access to fruit resources, such as *Saba senegalensis*, which is very important in their diet but also for the socio-economic activities of local communities, who harvest them for sale in major urban centres (Pruetz, 2006; Waller and Pruetz, 2016; Badji, 2019).

### 2.3 Collection and processing samples

The data collection phase took place from 22 February to 11 March 2022 in Fongoli. We tracked the group daily with the help of field assistants from 07:00 to 19:00 local time. We collected fresh faeces from 17 individuals, including 9 adult males, 3 young males, and 5 adult females. The collection was carried out in a non-invasive and opportunistic manner (Gillespie et al., 2008) after the faeces had been eliminated. Each sample was then fixed with 10 % formalin in sterile vials. In the case of doubt about the identity of an individual, no samples were taken to avoid confusion. This is because some individuals, particularly females and young animals, were more fearful and tended to blend in with their peers. In addition, the group frequently moved long distances during the day, and we had to keep a minimum distance of 10 m from the individuals, in accordance with the recommendations of Pruetz (2016). This limited the number of individuals that could be sampled, even though others were more accessible to us. Thus, 39 faeces samples from 17 individuals were collected for coproscopic analysis.

This analysis was carried out at the Interstate School of Veterinary Science and Medicine (EISMV) in Dakar (Senegal), using the sodium chloride (NaCl) flotation method and the sedimentation method to recover parasitic elements (Gillespie, 2006; Gillespie et al., 2008). For each faeces sample, three slides are prepared for parasite detection, and individuals are positive for a given parasitic taxon if it is observed in at least one sample of their faeces. Observation was carried out using an integrated camera optical microscope (Leica) with 10× and 40× objectives. Parasites were identified according to morphology, colour, size, and content, with the help of laboratory staff and plates (Howells et al., 2011; OMS, 2021). We calculated the prevalence for each parasite taxon, the average parasite richness in faeces samples, and the co-infection rate. We then compared the prevalence of each parasite (presence/absence) between males and females and between adults and juveniles using Fisher's exact tests from the “stats” package of the R studio software. To account for the repetitive nature of the collection, the sample size is limited to the 17 individuals whose stools were collected.

## 3 Results

**Figure 3:**
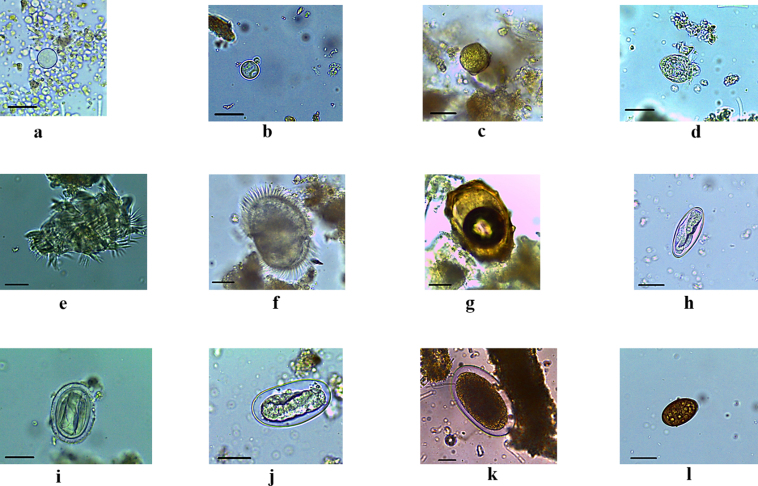
Diversity of gastrointestinal parasites (protozoa, helminths) identified in chimpanzee faeces. **(a)** *Entamoeba coli*, **(b)** *Entamoeba* spp., **(c)** Coccidia, **(d)** *Troglocorys* spp., **(e)** *Troglodytella* spp., **(f)** unidentified ciliate, **(g)** *Ascaris* spp., **(h)** *Enterobius* spp., **(i)** Spirurids, **(j)** *Strongyloides* spp., **(k)** Strongylids, **(l)** *Dicrocoelium* spp. Scale bar of panels **(a)**–**(l)**: 25 µ m.

Each sample contained at least one taxon of parasites. We found six taxa of protozoa (*Entamoeba* spp., *Entamoeba coli*, *Troglodytella* spp., *Troglocorys* spp., an unidentified ciliate, and Coccidia) and six taxa of helminth (*Ascaris* spp., *Enterobius* spp., *Strongyloides* spp., Spirurids, Strongylids, and *Dicrocoelium* spp.) (Table 1 and Fig. 3).

**Table 1:** Diversity and prevalence (%) of gastrointestinal parasites in chimpanzees at Fongoli ( N=17 ).

Parasite	Total ( n=17 )	Adult male ( n=9 )	Adult female ( n=5 )	Young male ( n=3 )
Protozoa	Prevalence (%)	Prevalence (%)	Prevalence (%)	Prevalence (%)
*Troglodytella* spp.	100	100	100	100
*Troglocorys* spp.	94	100	80	100
*Entamoeba* spp.	76	78	80	67
*Entamoeba coli*	29	22	40	33
Unidentified ciliate	59	67	20	100
Coccidia	94	100	80	100
Helminths				
*Enterobius* spp.	12	22	0	0
Spirurids	29	44	20	0
*Ascaris* spp.	12	11	20	0
*Strongyloides* spp.	47	44	20	100
Strongylids	18	11	0	67
*Dicrocoelium* spp.	12	11	0	33

N : total number of total individuals in the sample. n : number of individuals per category.

We recorded an overall prevalence of 100 % for protozoa and 70 % for helminths. All individuals hosted between four and eight parasite taxa, with variations ranging from two to six protozoa and zero to three helminths present. On average, there were 6 ± 1.41 parasitic taxa per individual, including 4.53 ± 1.07 protozoa per individual and 1.47 ± 1.07 helminths per individual. Additionally, individuals exhibited co-infection rates of 100 % for protozoa and 76 % for helminths. We identified six protozoa and six helminths in males and adults, compared to six protozoa and three helminths in females and juveniles (Table 1). Fisher's test indicated that there are no differences between sex or age categories (Fisher's exact test, p=1 in each case).

## 4 Discussion

Chimpanzees in Fongoli have been the focus of studies aimed at better understanding the diversity and prevalence of intestinal parasites affecting them (Howells et al., 2011; Ba, 2018; Bakhoum et al., 2021). The number of identified taxa (six protozoa, four nematodes, and one digenean) remains within the same range of parasitic diversity as reported by Howellset al. (2011): six nematodes, one cestode, and five protozoa; Ba (2018): six protozoa, four nematodes, and one cestode; and Bakhoum et al. (2021): six nematodes and one digenean. This suggests a relative stability in the community of gastrointestinal parasites affecting chimpanzees in Fongoli.

Chimpanzees exhibit co-infection rates of 100 % with protozoa and 76 % with helminths, hosting an average of 6 ± 1.41 parasite taxa per individual. This is notably higher than the 3.3 taxa reported in chimpanzees from Uganda (McLennan et al., 2017) living in anthropogenic landscapes. However, when considering only the helminth group, the average number of identified parasites (1.47 ± 1.07 helminths per individual) is quite similar to the rate reported by Ebbert et al. (2015) for chimpanzees in Niokolo-Koba National Park (1.6 ± 1.7 helminths per sample). It seems logical that chimpanzees in the park would be more exposed to encounters and exchanges of pathogens with sympatric mammals (e.g. baboons, warthogs) compared to their congeners in unprotected areas (Fongoli). In these environments, the density of these mammals may be reduced due to anthropogenic pressures such as poaching and agricultural practices (Pruetz, 2001).

Despite the extensive damage observed in the chimpanzees' habitat, we detected a high presence of entodiniomorph ciliates (*Troglodytella* spp., *Troglocorys* spp.). These organisms are considered essential components of the intestinal flora of chimpanzees, as they promote the digestion of fibre and polysaccharides (Pomajbíková et al., 2010, 2012).

Our results show a high prevalence of Coccidia infection in the chimpanzee group. This infection appears to be increasing compared to studies by Howells et al. (2011) and Ba (2018). These organisms are not usually detected in studies on gastrointestinal parasites in chimpanzees, even though some genera, such as *Cryptosporidium*, are known for their zoonotic potential (Roberts and Janovy, 2009). The increasing anthropisation of the environment in Fongoli through gold mining, timber trafficking, and seasonal grazing (Badji, 2019; Diallo et al., 2022) could promote Coccidia infection. These protozoa are associated with human environments and domestic animals, particularly ruminants. Livestock herds periodically frequent the chimpanzees' habitat during seasonal grazing lands. The faeces left by these animals can contaminate the soil and waterways and enable the faecal–oral transmission of Coccidia to various hosts, including chimpanzees.

We have identified amoebae, including *Entamoeba coli* and *Entamoeba* spp., which could correspond to the species *Entamoeba histolytica*/*dispar* due to the similarity of their cysts. Amoebae can infect several species of mammals and are often reported in non-human primate populations (Table 2). Their form of dissemination (cysts), which is highly resistant in the environment (Roberts and Janovy, 2009), could allow infections to persist for a long time in this community. Most amoeba species are considered non-pathogenic, with the exception of *Entamoeba histolytica*, which has been reported in sympatric baboons (Howells et al., 2011). This makes it highly likely that chimpanzees are already exposed to or carry this pathogen, which represents a threat to their conservation.

We found low prevalence rates for direct-cycle nematodes (*Ascaris* and *Enterobius*) and for the trematode *Dicrocoelium*. The prevalence rates for these three parasites are similar to those obtained in previous surveys (Table 2). Pinworms are regularly reported among intestinal parasites in non-human primates (Boundenga et al., 2021; N'da et al., 2022), unlike *Ascaris*, which are not commonly identified in wild chimpanzees. The presence of these parasites could be linked to the anthropisation of the environment and recurrent contact between humans, domestic animals, and wildlife. Their low prevalence could be explained by the high temperatures recorded in this type of Sudano-Guinean savannah ecosystem. The long months without rain, combined with the vast home range of savannah chimpanzees, considerably reduce the viability of infectious eggs in the soil and the likelihood of hosts encountering them. These helminths are therefore dependent on favourable conditions, particularly environmental humidity. The fluke identified is a perfect example of this, as the development of the larval stages depends on the availability of gastropods, which are inactive during the dry season (Sy et al., 2024). Furthermore, primates are not common hosts for this trematode. Infection could occur via domestic ruminants with a parasitic pattern similar to that of Coccidia and facilitated by the simultaneous presence of the first (gastropods) and second intermediate hosts (ants).

**Table 2:** Prevalence of identified parasites compared with reference studies.

Parasites identified	Study	Location	Prevalence (%)
Spirurids	This study	Fongoli, Senegal	29
	Howells et al. (2011)	Fongoli, Senegal	13.28
	Ba (2018)	Fongoli, Senegal	13.63
	Bakhoum et al. (2021)	Fongoli, Senegal	80
	Mcgrew et al. (1989)	Assirik, Senegal	31
	Laidoudi et al. (2020)	Dindifelo, Senegal	52.08
	Kalousova et al. (2014)	Ugalla, Tanzania	40.3
*Ascaris* spp.	This study	Fongoli, Senegal	12
	Howells et al. (2011)	Fongoli, Senegal	5.47
	Bakhoum et al. (2021)	Fongoli, Senegal	6.7
*Enterobius* spp.	This study	Fongoli, Senegal	12
	Ba (2018)	Fongoli, Senegal	4.54
	Boundenga et al. (2021)	Haut-Ogooué, Gabon	7.14
*Dicrocoelium* spp.	This study	Fongoli, Senegal	2
	Bakhoum et al. (2021)	Fongoli, Senegal	13.33
	McLennan et al. (2017)	Bulindi, Uganda	0.2
*Strongyloides* spp.	This study	Fongoli, Senegal	47
	Mcgrew et al. (1989)	Assirik, Senegal	21
	Bakhoum et al. (2021)	Haut-Ogooué, Gabon	66.7
	McLennan et al. (2017)	Bulindi, Uganda	57.9
	Howells et al. (2011)	Bulindi, Uganda	19.53
	Boundenga et al. (2021)	Haut-Ogooué, Gabon	67.14
*Troglodytella* spp.	This study	Fongoli, Senegal	100
	Howells et al. (2011)	Fongoli, Senegal	64.84
	Ba (2018)	Fongoli, Senegal	77.27
	McGrew et al. (1989)	Assirik, Senegal	77
	McLennan et al. (2017)	Bulindi, Uganda	79.6
	Kalousova et al. (2014)	Ugalla, Tanzania	62.2
	Boundenga et al. (2021)	Haut-Ogooué, Gabon	7.14
*Troglocorys* spp.	This study	Fongoli, Senegal	94
	Howells et al. (2011)	Fongoli, Senegal	60.16
	Ba (2018)	Fongoli, Senegal	9.09
	McLennan et al. (2017)	Bulindi, Uganda	15.3
	Kalousova et al. (2014)	Ugalla, Tanzania	19.3
*Entamoeba* spp.	This study	Fongoli, Senegal	76
	McLennan et al. (2017)	Bulindi, Uganda	39.1
	Kalousova et al. (2014)	Ugalla, Tanzania	6.7
	Boundenga et al. (2021)	Haut-Ogooué, Gabon	47.86
*Entamoeba coli*	This study	Fongoli, Senegal	29
	Howells et al. (2011)	Fongoli, Senegal	10.16
	McLennan et al. (2017)	Bulindi, Uganda	8.3
Coccidia	This study	Fongoli, Senegal	94
	Ba (2018)	Fongoli, Senegal	22.72

*Strongyloides* spp. and Spirurid nematodes were the most common in chimpanzees. Spirurids may belong to the Physalopteridae family reported in previous studies on chimpanzees in Senegal. The high prevalence rates obtained in these various studies suggest that these helminths are well adapted to these environments (Table 2). Their complex life cycles may favour their establishment in these primates. Their infectious stages do not appear to be directly dependent on environmental factors in the same way as direct-cycle nematodes. Infection with *Strongyloides* can occur through active penetration of the skin by infectious larvae or through autoinfection within the host (Roberts and Janovy, 2009). In the case of Spirurids, the larvae first develop in intermediate hosts (beetles, mites) or paratenic hosts (*Galago senegalensis*) before passing to chimpanzees through feeding (McGrew, 1983; Pruetz, 2006; Roberts and Janovy, 2009).

Parasite diversity and prevalence were compared according to age and sex (Table 1). We identified three helminths in females compared to six in males, with higher prevalence in males. The same pattern was observed according to age, with three helminths observed in juveniles compared to six in adults. Protozoa were found in all subgroups with high prevalence rates. Statistical analysis showed no significant difference between males and females ( p=1 ) or between adults and young individuals ( p=1 ) for either protozoa or helminth prevalence. Furthermore, these results must be put into perspective, taking into account the small sample size and the predominance of adult males. The latter form the most accessible category of the group for non-invasive faecal collection. One solution could be to expand the study to include other chimpanzee groups. However, it may be difficult to obtain data on the demographic composition of unaccustomed groups or sampled individuals. The study period could also be extended to cover the rainy season in order to determine the influence of seasons on the diversity and prevalence of gastrointestinal parasites.

In summary, the study identified 12 taxa of gastrointestinal parasites, with a high prevalence of protozoa in samples collected from Fongoli chimpanzees. Our results show a potential increase in the prevalence of parasites closely associated with humans and domestic animals based on previous surveys conducted on the same group. It is therefore recommended that local populations be made aware of the importance of preserving these pockets of habitat for chimpanzees and other sympatric species. In addition, it is essential to monitor the health of humans and domestic animals and to regulate certain practices such as grazing and forest resource exploitation in order to reduce the exchange of pathogens.

## Data Availability

All data generated by this study appear in the article.
